# Role of hypoxia-inducible factor in postoperative delirium of aged patients: A review

**DOI:** 10.1097/MD.0000000000035441

**Published:** 2023-09-29

**Authors:** Hu Shen, Jianyin Yang, Xu Chen, Yu Gao, Baoming He

**Affiliations:** a Department of Neurology, Sichuan Provincial People's Hospital, School of Medicine, University of Electronic Science and Technology of China, Chengdu, China; b Department of ICU, Chengdu Xinjin District Hospital of Traditional Chinese Medicine, Chengdu, China; c Department of Pharmacy, Chengdu Women’s and Children’s Central Hospital, School of Medicine, University of Electronic Science and Technology of China, Chengdu, China.

**Keywords:** anesthesia, elderly, HIF, postoperative delirium

## Abstract

Postoperative delirium is common, especially in older patients. Delirium is associated with prolonged hospitalization, an increased risk of postoperative complications, and significant mortality. The mechanism of postoperative delirium is not yet clear. Cerebral desaturation occurred during the maintenance period of general anesthesia and was one of the independent risk factors for postoperative delirium, especially in the elderly. Hypoxia stimulates the expression of hypoxia-inducible factor-1 (HIF-1), which controls the hypoxic response. HIF-1 may have a protective role in regulating neuron apoptosis in neonatal hypoxia-ischemia brain damage and may promote the repair and rebuilding process in the brain that was damaged by hypoxia and ischemia. HIF-1 has a neuroprotective effect during cerebral hypoxia and controls the hypoxic response by regulating multiple pathways, such as glucose metabolism, angiogenesis, erythropoiesis, and cell survival. On the other hand, anesthetics have been reported to inhibit HIF activity in older patients. So, we speculate that HIF plays an important role in the pathophysiology of postoperative delirium in the elderly. The activity of HIF is reduced by anesthetics, leading to the inhibition of brain protection in a hypoxic state. This review summarizes the possible mechanism of HIF participating in postoperative delirium in elderly patients and provides ideas for finding targets to prevent or treat postoperative delirium in elderly patients.

## 1. Introduction

Postoperative delirium is common, especially in older patients, with an incidence that varies widely depending on the patient population and type of surgery. According to reports, the incidence of delirium is 15% to 35% in patients over the age of 65 within 2 weeks after surgery.^[1]^ Delirium is associated with prolonged hospitalization, increased morbidity, and significant mortality. Several pathophysiological mechanisms may contribute to delirium onset, including neurotransmitter imbalance, systemic inflammatory response syndrome, and neuroinflammation,^[1–3]^ altered brain metabolism, and impaired neuronal network connectivity.^[4–9]^ There is currently no convincing evidence that any prophylactic measure prevents postoperative delirium because the mechanism of postoperative delirium is not clear.

When monitoring rSO_2_ in elderly patients following noncardiac abdominal surgery, cerebral desaturation (hypoxia) has been noted in more than 20% of instances as one of the independent risk factors for postoperative delirium.^[4–6]^

Hypoxia stimulates the expression of hypoxia-inducible factor-1 (HIF-1), which controls the hypoxic response.^[10–15]^ HIF-1 may have a protective role in regulating neuron apoptosis in neonatal hypoxia-ischemia brain damage and may promote the repair and rebuilding process in the brain that was damaged by hypoxia and ischemia.^[10]^ Animal experiments show that neuron-specific loss of HIF-1 increases ischemic brain injury, and the recovery of neural function in ischemic rats is related to the increased expression of hypoxia-inducible factor subtype 1α (HIF-1α).^[10,11]^ And more recently, anesthetics and perioperative drugs have been reported to affect HIF-1 activity.^[16–20]^ Research reports that the anesthetics inhibit HIF-1 activation and downstream gene expression.^[13–18]^ Hippocampal HIF-1α/vascular endothelial growth factor (VEGF) signaling seems to be the upstream mechanism of isoflurane-induced cognitive impairment and provides a potential preventive and therapeutic target for postoperative delirium.^[16]^

So, we hypothesize that HIF plays an important role in the pathological process of postoperative delirium in the elderly. This review summarizes the possible mechanism of HIF participating in postoperative delirium in elderly patients and provides ideas for finding targets to prevent or treat postoperative delirium in elderly patients.

## 2. Anesthesia causes cerebral hypoxia, resulting in postoperative delirium

Anesthetics can cause cerebral hypoxia, and the degree of cerebral hypoxia is related to the type of anesthetic. Research confirms that cerebral desaturation occurs during general anesthesia.^[17]^ Cerebral desaturation occurs in nearly 20% of elderly patients undergoing major abdominal nonvascular surgery, resulting from a multicenter, prospective, randomized, and blinded study.^[8]^ Cerebral desaturation occurred during ordinary general anesthesia and might be associated with the use of anesthetics.^[12]^ Different anesthetics have different effects on cerebral oxygen metabolism; cerebral desaturation during cardiopulmonary bypass was more frequent in the fentanyl group than in the propofol group.^[19]^ The cause of brain hypoxia caused by anesthetics is related to their cardiovascular inhibition. All general anesthetics produce cardiovascular depression that can be augmented in the elderly patient, potentially exposing him or her to inadequate brain perfusion.^[20,21]^ Inadequate brain perfusion leads to cerebral hypoxia.

Many studies have confirmed the correlation between postoperative delirium and cerebral oxygen metabolism. Intraoperative cerebral oxygen desaturation was reported to be associated with postoperative cognitive dysfunction^[22–24]^ and early postoperative neuropsychological dysfunction^[25]^ in elderly patients, especially when anesthetics and sedatives are combined.^[26]^ However, the type of anesthetic used does not influence the incidence of postoperative cognitive dysfunction.^[27]^ Cerebral hypoxia might be involved in the physiopathology of cognitive decline observed after surgery in the elderly patient.^[19]^

According to relevant reports, improving cerebral oxygen metabolism may be beneficial to reduce postoperative delirium. Elderly individuals undergoing spine surgery in the prone position may benefit from lung-protective ventilation due to a mechanism involving lowered inflammatory responses and enhanced cerebral oxygen metabolism.^[28]^

## 3. Roles of HIF in cerebral hypoxia

The brain is one of the main organs of the body that strongly respond to acute hypoxia.^[29]^ Hypoxia stimulates the expression of many genes, and 1 key protein involved is HIF-1, which is a transcription factor that regulates adaptive responses to the lack of oxygen in mammalian cells. HIF-1 consists of 2 proteins, HIF-1 alpha and HIF-1 beta. HIF-1alpha accumulates under hypoxic conditions, whereas HIF-1beta is constitutively expressed.^[30]^ HIF-1alpha is immunologically detectable in the few minutes following low PO_2_ exposure. Besides HIF-1, HIF-2, and HIF-3 also play a role in the adaptive response to hypoxia. The difference is as follows: HIF-1 is more essential for the regulation of adaptation to hypoxia in neurons, and HIF-2α is more important for the endothelium of microvessels.^[31]^ HIF-3α serves as an endothelial cell fate executor during chronic hypoxia.^[32]^

The protective effects of HIF-1 in the brain have been confirmed by several studies. The HIF signaling pathway plays an important role in intrinsic neuroprotection. HIF-1α expression increased the brain hypoxia adaptation capability of the rat through the expression of genes.^[33]^ Upregulation and maintenance of HIF-1α expression may attenuate vascular cognitive impairment.^[34]^ HIF-1alpha may have a protective role in regulating neuron apoptosis in neonatal hypoxia-ischemia brain damage and may promote the repair and rebuilding process in the brain that was damaged by hypoxia and ischemia.^[35]^ HIF-1α is involved in the neurodegeneration induced by isoflurane in the brain of neonatal rats and has been found to regulate both prosurvival and prodeath pathways in the CNS.^[5]^ Maintaining the HIF-1α level by inhibiting the prolyl 4-hydroxylase was effective in attenuating the nerve damage during hypoxia and postponing the incidence of Alzheimer disease.^[36]^ Hippocampal apoptosis increased and cognitive function deteriorated when HIF-1α was inhibited. HIF-1α has a neuroprotective effect in subarachnoid hemorrhage.^[37]^

## 4. Potential mechanisms by which HIF-1 protects brain neurons

As mentioned above, HIF-1 plays a protective role in brain injury. How does HIF-1 participate in the pathological process of postoperative delirium? In this section, we reviewed the potential regulatory mechanisms of HIF-1 in postoperative delirium.

### 4.1. HIF-1 and inflammatory mediators

Several pathophysiological mechanisms may contribute to delirium onset. As a key hallmark of neurological complications, inflammation plays a key pathogenic role in the development of postoperative delirium,^[5,36–41]^ including neuroinflammation and the immune-inflammatory response system.^[38]^ Following hip fracture and surgery, delirium and psychomotor problems may be brought on by immune-inflammatory and oxidative stress pathways, most often as a result of an aseptic inflammatory process.^[39]^ The release of pro-inflammatory cytokines and chemokines by activated glial cells, astrocytes, and microglia plays an important role in the neuroinflammatory process. Research shows patients who suffer from delirium are accompanied by significantly increased numbers of white blood cells, neutrophil percentage, neutrophil/lymphocyte ratio, and lower mean platelet volume.^[40]^ IL-6 seems to be a consistent predictor of delirium in surgical samples.^[41]^ The elevated plasma IL-6 levels are signatures of poststroke delirium.^[41]^ IL-8 levels are associated with delirium onset, and underlying depression or dementia influences IL-8 levels.^[42]^ In patients with delirium, higher levels of pro-inflammatory cytokines and cortisol were identified in plasma and cerebrospinal fluid.^[43]^ Perioperative cortisol and inflammatory alterations observed in mild cognitive impairment may provide a physiological explanation for this increased risk of mild cognitive impairment.^[44]^ Mild cognitive impairment is associated with a higher risk of postoperative delirium.

HIF-1α has a prominent anti-inflammatory role in the acute inflammatory processes of multiple diseases.^[45]^ Many genes could be involved in HIF-1α regulation, including MAPKs, *miR-155-5p*, and so on.^[46]^ Research shows *miR-155-5p* directly targets HIF-1α and negatively regulates its expression. Inhibition of *miR-155-5p* enhanced cell viability and prevented cell apoptosis, significantly decreased infarct volume, improved neurobehavioral outcomes in middle cerebral artery occlusion (MCAO) rats, inhibited inflammation and oxidative stress, and resulted in enhanced protection against cerebral infarction after NSC transplant.^[46]^ HIF-1α can sufficiently control the progression of neurological symptoms after an ischemic stroke owing to the actions associated with its downstream genes.^[46]^

And more, HIF-1α with a decline in iNOS, tumor necrosis factor-α (TNF-α), and NF-kB levels, implying the anti-inflammatory role of HIF-1α activator following stroke.^[47]^ Intraperitoneal injection of the HIF inhibitor, acriflavine hydrochloride, abolished the protection of RIPC with respect to infarct size and neurological functions and neutralized the downregulation of pro-inflammatory IL-1, IL-6, and IFN-γ.^[48]^ DEX-regulated HDAC2 may play an inhibitory role in mice with POCD through regulation of the HIF-1α/PFKFB3 axis.^[49]^ HIF-1α activated in diabetic retina is likely to play a role in regulating pathophysiological processes via IL-6 and TNF-α mechanisms. This has pharmacological implications to target specific HIF-1, IL-6, and TNF-α signaling pathways for dysfunction and vulnerability related to DR.^[50]^ Dex promotes the recovery of renal function and reduces the inflammatory level in RIRI rats through the PI3K/Akt/HIF-1α signaling pathway.^[51]^ HIF-1α was overexpressed in COPD, which upregulated expressions of inflammatory factors via activating the EGFR/PI3K/AKT pathway. The activated EGFR/PI3K/AKT pathway induced by pulmonary inflammation further upregulated HIF-1α expression in a feedback loop, thus aggravating COPD pathological changes.^[52]^ HIF-1α participates in the inflammatory response process caused by Ang II and downregulation of HIF-1α may be able to partially protect or reverse inflammatory injury in podocytes.^[53]^ The increased activity of HIF-α isoforms regulates Th1/Th17 mediated inflammation in sarcoidosis.^[54]^ Inflammatory responses of endothelial cells to hypoxia with concurrent acidosis are dynamically regulated by the combined actions of hypoxia, miR-126, and HIF-1*α* on the master regulator high-mobility group box-1.^[55]^ There are 3 classical inflammatory pathways: p38 MAPK, IL-6/JAK/STAT3, and PI3K; and a nonclassical inflammatory pathway, the Hippo. Recently, the Hippo pathway has been linked to various inflammatory modulators such as FoxO1/3, TNFα, IL-6, COX_2_, HIF-1α, AP-1, JAK, and STAT.^[56]^ Troxerutin could regulate HIF-1α by activating JAK2/STAT3 signaling to inhibit oxidative stress, inflammation, and apoptosis of cardiomyocytes induced by H_2_O_2_.^[57]^HIF-1 reduced inflammation in spinal cord injury via miR-380-3p/ NLRP3 by Circ 0001723.^[58]^ prolyl hydroxylase (PHD)-1 downregulation skews the hypoxic response toward enhanced protective HIF-1α stabilization in the inflamed mucosa of UC patients.^[59]^

Cytokines also influence HIF signaling. Malkov et al^[60]^ report extensive evidence for TNF-α and interleukin-1β directly impacting HIF signaling through the regulation of HIF at transcriptional and posttranslational levels. The pathophysiology of delirium in older adults is complex, and inflammation is a relevant precipitant factor in this syndrome. The release of pro-inflammatory cytokines and chemokines by activated glial cells, astrocytes, and microglia plays an important role in the neuroinflammatory process. HIF-1α reduces the level of inflammatory factors through the regulation of signal pathways and plays an anti-inflammatory role. The inhibition of HIF-1 may increase the inflammatory response and cause postoperative delirium.

## 5. HIF-1 and oxidative stress

Abnormally activated oxidative stress might be involved in the underlying mechanisms of postoperative delirium.^[61–64]^ Disturbed serotonergic neurotransmission and an increased status of oxidative stress in patients with delirium.^[63]^ Patients with low preoperative catalase levels appeared to be more susceptible to delirium than patients with higher catalase levels.^[64]^

Several studies have reported that HIF-1α affects the oxidative stress response through different mechanisms. HIF-1α protects against oxidative stress by directly targeting mitochondria.^[59]^ Mitochondrial ferritin (a protein [FtMt]) can alleviate hypoxia-induced brain cell death by sequestering uncommitted iron and preventing oxygen-derived redox damage.^[65]^ HIF-1α can upregulate FtMt expression to prevent oxygen-derived redox damage.^[66]^ During cerebral ischemia/reperfusion injury-induced lung injury, the body may upregulate antioxidative stress activities and promote angiogenesis to repair the endothelial barrier through the Nrf2/HO-1 and HIF-1α/VEGF signaling pathways, enabling self-protection.^[67]^ One of the proteins induced by HIF, EPO, has the properties of being antiapoptotic, antioxidant, and protective for neurons, astrocytes, and oligodendrocytes.^[16]^ A novel HIF stabilizer, FG4592 (Roxadustat), enhanced renal vascular regeneration, possibly via activating the HIF-1α/VEGFA/VEGF receptor 1 (VEGFR1) signaling pathway and driving the expression of the endogenous antioxidant superoxide dismutase 2 (SOD2).^[68–70]^

### 5.1. HIF-1 and energy metabolism

Brain functioning and high-order cognitive functions critically rely on glucose as a metabolic substrate. Acutely reduced glucose metabolism impairs cognition selectively in the vulnerable brain.^[69]^Disrupted brain energy metabolism may be associated with postoperative delirium.^[70–74]^

HIF-1 plays a very important role in energy metabolism. Research shows that under conditions of hypoxia, most eukaryotic cells can shift their primary metabolic strategy from predominantly mitochondrial respiration towards increased glycolysis to maintain ATP levels. Aerobic glycolysis is also a key regulator of synaptic plasticity in the brain that may positively influence cognition.^[72]^ HIF-1a activation represents a well-characterized mechanism by which the cell can quickly respond to hypoxic environments by upregulating glycolysis and inhibiting mitochondrial respiration in order to meet cellular energy demands and contribute to cell survival in hypoxic tissues.^[73–77]^ Hif-1α transcription was upregulated *via* the mTORC1/eIF4E pathway to drive glycolysis.^[78–80]^ The increase of glucose transport activity is achieved by inducing GLUT1 and GLUT3 expression and upregulating HIF-1α expression.^[81]^

## 6. Anesthetics affect HIF-1 activity in elderly patients.

Intraoperative cerebral oxygen desaturation occurred during general anesthesia and was associated with postoperative cognitive dysfunction.^[20,21,79–81]^ Cerebral cellular responses to hypoxia are associated with a family of transcription factors called HIFs, which induce the expression of a wide range of genes that help cells adapt to a hypoxic environment. HIF-1 is a heterodimer comprising α and β subunits.^[80]^ Under normoxic conditions, the β subunit is constitutively expressed in the cell, while the α-subunit undergoes rapid ubiquitin-dependent proteasome degradation due to the action of oxygen dependent HIF PHD.^[81]^ Under hypoxic conditions, PHD is inactivated, leading to the stabilization of HIF-1α, followed by its translocation into the nucleus, where it forms a heterodimeric complex with HIF-1β.^[20]^ This complex interacts with DNA and activates the expression of multiple target genes encoding proteins that influence neuroinflammation, the immune-inflammatory response system, oxidative stress, and enzyme metabolism. The HIF-1 signaling pathway protects brain tissue through the above mechanisms. In adults, even when intraoperative cerebral oxygen desaturation occurs during general anesthesia, HIF complex activation initiates a neuroprotective response, resulting in the restoration of cellular functions that are affected by hypoxia.

However, an age-dependent decline in cortical HIF-1α accumulation and activation of HIF target genes in response to hypoxia.^[21,22]^ Thus, HIF-1α levels and recovery speed are much lower in the elderly when responding to hypoxia than in the young. Several articles have confirmed this idea. Under hypoxic stimulation, HIF binding to DNA increased in the young and middle-aged rats but not in the old rats.^[82]^ HIF-1α and fetal liver kinase 1 levels were lower in old rats than in young rats.^[83]^ Inhibition of HIF-1 transactivation of gene expression in the young brain recovering from MCAO. Furthermore, a copper metabolism MURR domain 1 (COMMD1) was significantly elevated after MCAO only in the brains of aged rats, and suppression of COMMD1 by siRNA targeting COMMD1 restored HIF-1 transactivation and improved recovery from MCAO-induced damage in the aged brain. Ndubuizu OI^[84]^ reported that HIF-1α accumulation and transcriptional activation of HIF target genes in aged rats are significantly attenuated during acute hypoxic exposure. Cortical HIF-1α accumulation and HIF-1 activation remain absent during chronic hypoxic exposure in the aged rat brain. A paper suggests a compensatory HIF-1-independent preservation of hypoxic-induced microvascular angiogenesis in the aged rat brain.^[85]^ HIF-1α accumulation was attenuated, and VEGF expression was decreased in the cerebral cortex of aged mice. The HIF-1 response to hypoxia is greatly attenuated, leading to an initial delay in the cerebral angiogenic response in aged mice in the first week of hypoxia. The delayed adaptive response, however, may result in decreased survival in the older cohort.^[86]^ Following injury, in the aged animals, the increase in HIF-1alpha and VEGF in response to injury was much lower than in the adult injured animals. These results support the hypothesis that reduced expression of genes in the HIF-1alpha neuroprotective pathway in aging may contribute to poor prognosis in the elderly following traumatic brain injury.^[87]^ Impaired HIF-1 transcription activity is associated with defective cerebral recovery from ischemic stroke in aged rats.^[88]^ Impaired HIF-α may contribute to age-associated cognitive decline during hypoxic events.^[85]^

**Figure 1.**
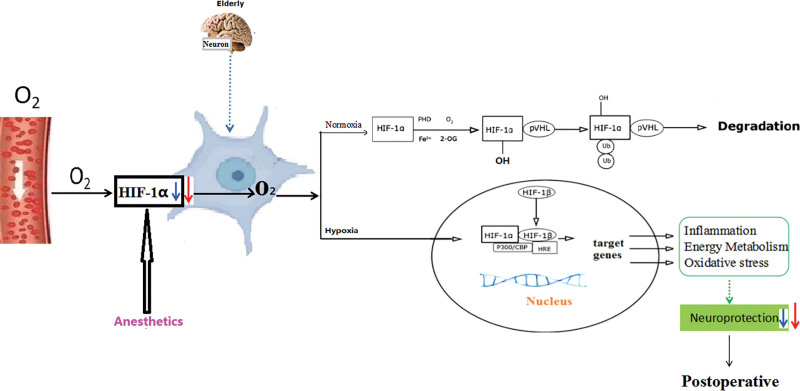
A hypothetical model for the pathophysiological relationship between delirium and hypoxia-inducible factor-1 (HIF-1) in the elderly. (A) Hypoxia stimulates the expression of HIF-1, which controls the hypoxic response. (B) HIF-1α levels and recovery speed are much lower in the elderly when responding to hypoxia than those of the young. (C) During general anesthesia, anesthetics inhibited HIF-1α protein neosynthesis and HIF-1α activation in the aged patient brain, further aggravating the HIF-1 response to hypoxia, which led to a decreased neuroprotective effect. HIF-1α = hypoxia-inducible factor subtype 1α.

Anesthetics and perioperative drugs have been reported to affect HIF-1 activity (Fig. 1). Research reports that the anesthetics inhibited lipopolysaccharide-induced HIF-1alpha expression. HIFalpha-hydroxylases activity and HIF-1alpha stability were not affected, but the HIF-1alpha protein synthesis was inhibited by the anesthetics.^[89]^ The intravenous anesthetic propofol inhibited HIF-1α activation induced by hypoxia or CoCl^2^. Propofol also prevented isoflurane-induced HIF-1α activation, and partially reduced cancer cell malignant activities.^[14,90]^ Sevoflurane can reduce the content of inflammatory factors, inhibit apoptosis, and reduce the expressions of HIF-1 and HSP70 in the case of cerebral ischemia/reperfusion injury ischemia/reperfusion injury.^[90]^ In addition, propofol, a general anesthetic, was found to significantly reduce the LPS-induced upregulation of HIF-1α and ROS in a dose-dependent manner.^[91]^ Results from the Transwell assay confirmed that propofol also suppressed cell invasion by decreasing HIF-1α expression in the LPS-treated NSCLC cells.^[92]^ Further, propofol suppressed Hif-1*α* expression by inhibiting the upregulation of NF-*κ*B p65 after exposure to hypoxia in BV2 microglia. Propofol attenuates hypoxia-induced neuroinflammation, at least in part by inhibiting oxidative stress and NF-*κ*B/ Hif-1*α*

signaling.^[93]^ Propofol but not sevoflurane limits HIF-1α activation in hepatic ischemia/ reperfusion injury.^[94]^ Propofol attenuates intracellular Ca^2+^ concentration, CaMKII and AKT phosphorylation, and HIF-1α expression, probably via inhibiting the NMDA receptor, thus inhibiting glycolysis and adhesion of tumor and endothelial cells.^[95]^

## 7. Conclusion

From the above studies, we speculate that during general anesthesia, anesthetics inhibited HIF-1α protein neosynthesis and HIF-1α activation in the aged patient’s brain, further aggravating the HIF-1 response to hypoxia, which is greatly attenuated and delayed in the brain of the elderly. Impaired HIF-α induced by anesthetics in the elderly may contribute to postoperative delirium. It is possible to provide a new mechanism of neuronal injury induced by anesthetics and postoperative delirium.

## Author contributions

**Conceptualization:** Hu Shen.

**Data curation:** Xu Chen, Yu Gao.

**Methodology:** Jianyin Yang.

**Writing – original draft:** Hu Shen, Jianyin Yang, Xu Chen.

**Writing – review & editing:** Baoming He.
